# Femtosecond laser hybrid processing strategy of transparent hard and brittle materials

**DOI:** 10.3389/fchem.2022.1082738

**Published:** 2022-11-24

**Authors:** Jia-Wei Tan, Gong Wang, Guo-Xu Zhao, Ya-Chong Hou, De-Rong Sun, Yi-Fei Song, Le-Yan Dong, Hui Zhao, Yulei Wang

**Affiliations:** ^1^ Center for Advanced Laser Technology, Hebei University of Technology, Tianjin, China; ^2^ Hebei Key Laboratory of Advanced Laser Technology and Equipment, Tianjin, China; ^3^ School of Information and Electrical Engineering, Hebei University of Engineering, Handan, China

**Keywords:** femtosecond laser, 3D processing, micro-nano fabrication, transparent hard and brittle materials, hybrid processing strategy

## Abstract

With high hardness, high thermal stability, chemical inertness and excellent optoelectronic properties, transparent hard and brittle materials have drawn significant attentions in frontier domains such as aerospace, photoelectric detection, and high-intensity lasers. Femtosecond laser processing technology demonstrates great potential for transparent hard and brittle materials processing due to its outstanding advantages such as non-contact, true 3D processing and programmable design. However, high-energy laser ablation usually causes severe damage to the surface of the materials, resulting in low processing accuracy, low processing efficiency and poor surface quality. Femtosecond laser hybrid processing strategies have been proven to be an effective solution to solve the above problems. This mini-review summarizes the fundamentals and research progress of femtosecond laser hybrid processing strategies of transparent hard and brittle materials in recent years. Moreover, the challenges and application prospects of these techniques are discussed.

## Introduction

The rapid development of optoelectronics, nanophotonics, biomanufacturing and bionics has placed higher demands on the fabrication of micro/nanodevices. Transparent hard and brittle materials, such as diamond and sapphire, have become ideal choices for micro/nano devices operating under harsh environmental conditions such as strong radiation and easy corrosion, due to their high hardness, high thermal stability, chemical inertness, and broadband transparency (Khattak et al., 2016). However, its high hardness, high stability and other characteristics make it difficult to achieve precision processing by traditional processing techniques. For example, nano-embossing and thermal transfer technologies are not suitable for high hardness and high temperature-resistant materials, since the transfer process is easy to lead to the formation of chipping, cracking, and other stress damage. Femtosecond laser processing technology, as a new micro-nanofabrication tool, has shown great potential for micro-nano processing of transparent hard and brittle materials with the advantages of high processing accuracy, flexibility, contactless processing with no material selectivity (Chen et al., 2018; Sima et al., 2018; Lin and Hong, 2021). It enables flexible and efficient three-dimensional (3D) fabrication of submicron feature sizes and has a wide range of applications in the fabrication of 3D micro/nanostructures in the fields of micro-optics, micro-fluidics, super-impregnated functional surfaces, and bionic micro-robotics, which drives the development of functionalization, miniaturization, and integration of complex devices (Chen et al., 2012).

Tightly focused femtosecond laser has a high-power density. The sharp increase in temperature through electron collisions generated by inverse bremsstrahlung absorption and subsequent electron-lattice interactions leads to ablation and removal of material in and around the focused center of the spot. As a result, laser ablation has also become one of the most promising general laser processing technologies (Li et al., 2022; Liu S.-F et al., 2022). Femtosecond laser ablation techniques usually use a high power density above the material damage threshold to achieve material removal. Actually, material ablation removal is a non-equilibrium process. In the case of high-energy laser ablation, the ablated surface morphology changes. A large number of debris and particles are produced, which causes light scattering and hinders in subsequent process. The degree of damage to the material can be reduced by regulating the processing parameters. However, the inherent hardness, brittleness, and low light absorption of transparent hard and brittle materials make the processed surface quality and the processing efficiency still cannot meet the high requirements in micro-optics and microfluidics. Various femtosecond laser hybrid processing strategies have been proposed to address these challenges, providing many practical solutions for high-quality and high-precision processing of transparent hard and brittle materials.

In this mini-review, we summary the recent research advances in femtosecond laser hybrid processing strategies of transparent hard and brittle materials, focusing on three processing strategies: femtosecond laser-assisted etching strategy, liquid-assisted femtosecond laser ablation strategy, and femtosecond laser combined with annealing strategy. Moreover, this mini-review briefly discusses the challenges of femtosecond laser hybrid processing strategies and provides an outlook for the future.

### Femtosecond laser-assisted etching strategy

Femtosecond laser-assisted etching strategy effectively improves the surface quality of transparent hard and brittle materials after femtosecond laser processing (Liu et al., 2019a). The basic principle is that the material is modified by femtosecond laser to induce a phase change or compositional change, which results in a different etching rate in the modified region and unmodified region. The modified areas can be removed during the subsequent etching process by controlling the etching parameters (Wang et al., 2022a). Ultimately, micro/nanostructures can be created on the surface or inside transparent hard and brittle materials. The etching process used is mainly divided into wet etching and dry etching.

Marcinkevičius et al. (2001) were the first to propose femtosecond laser-assisted wet etching and demonstrated direct 3D micromachining inside silica. It is worth noting that this technology allows the fabrication of 3D channels with diameters down to 10 mm in volume and with arbitrary interconnection angles and high aspect ratios. This pioneering work has demonstrated the feasibility of the strategy. However, the mechanism leading to the different etching rates is unclear. Researchers conducted extensive studies on the femtosecond laser-modified region and etching rate. For example, Bellouard et al. (2004) observed that the central portion of the laser processing path etched faster and explained the change in etching rate due to femtosecond laser modification. They suggest that the increase in etch rate is caused by two mechanisms: one by the presence of internal stresses and the other by a decrease in the average ring size of the structure due to changes in the crystalline state. This provides new insights into the laser-matter interaction. By ultra-high spatial resolution measurements, Hnatovsky et al. (2006) demonstrated that the difference in etching rates was dependent on the presence of polarization-dependent self-ordered periodic nanopores or nanopore structures. In addition, they investigated the optimal processing conditions for preparing high-quality microchannels, which had led to the development of 3D monolithic integration of microchannels and microphotonic assemblies. Mazilu et al. (2007) performed structural characterization of laser-modified regions inside sapphire. The results revealed the presence of dislocations near the sapphire crystal-amorphous boundary after femtosecond laser processing; while the high density of dislocations did not affect the etching ability of sapphire in aqueous hydrofluoric acid solutions. The luminescence and Raman characterization of the femtosecond laser-modified region were analyzed (Choudhury et al., 2013). It was demonstrated that femtosecond laser pulses transformed the neodymium-doped yttrium aluminum garnet (Nd:YAG) crystal state into a pre-damaged, which in turn showed a greater etching rate than that of the unmodified region.

The above-mentioned mechanistic studies lay a solid foundation for femtosecond laser-assisted wet etching to prepare micro/nanostructures. Self-organized nanostructures, elliptical microchannels and concave microstructures with smooth surfaces have been successfully prepared and applied in photonic crystals, biochemical analysis and super hydrophobicity (Wortmann et al., 2008; Hao et al., 2012; Bian et al., 2013). Shan et al. (2015) reported highly integrated on-chip 3D microcoil arrays inside fused silica, demonstrating the flexibility and versatility of femtosecond laser-assisted wet etching strategy. Wang et al. used temporally shaped femtosecond laser Bessel-beam-assisted chemical etching to achieve high throughput and high depth-to-diameter ratio microchannel preparation in fused silica (Wang et al., 2018). The etching depth was increased by 13 times with the temporally shaped Bessel beam modification compared with the conventional single pulse. In addition, Ródenas et al. (2019) demonstrated large-area 3D dense nanopore lattices prepared in yttrium aluminum garnet and sapphire crystals by wet etching-assisted laser direct writing techniques, respectively. They show that the wet etching rate can be increased by 5 orders of magnitude, making it possible to prepare arbitrary 3D structures with feature sizes of 100 nm.

**FIGURE 1:**
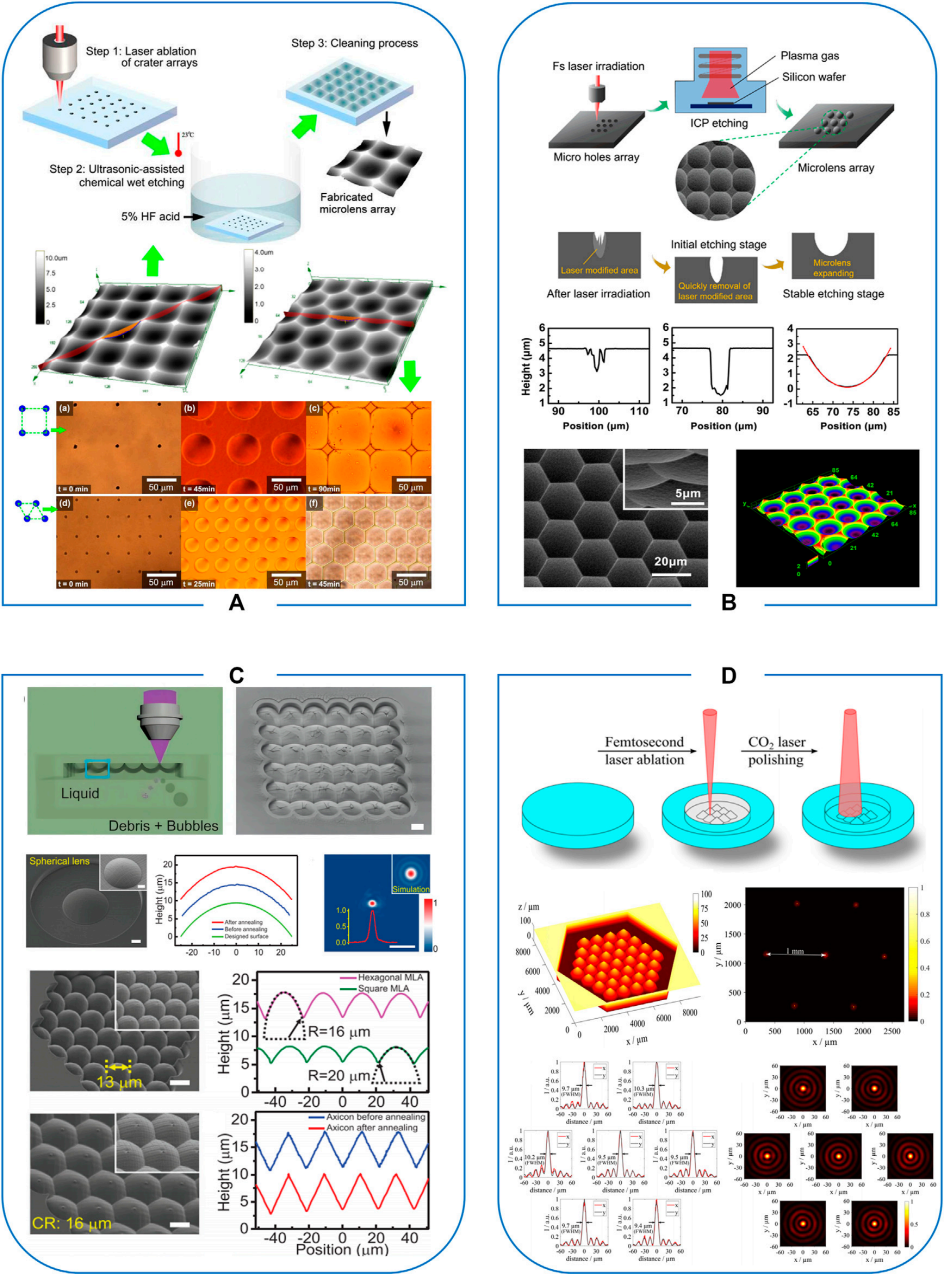
**(A)** Femtosecond laser-assisted wet etching; reproduced with permission from Chen et al. (2010). **(B)** Femtosecond laser-assisted dry etching; reproduced with permission from Liu et al. (2017). **(C)** Liquid-assisted femtosecond laser ablation; reproduced with permission from Hua et al. (2022). **(D)** Femtosecond laser combined with annealing; reproduced with permission from Schwarz et al. (2020).

In addition to processing micropore and microchannel structures (Juodkazis et al., 2008), microlens arrays (MLAs) of various shapes with good optical properties can be prepared by femtosecond laser-assisted wet etching strategy. As shown in Figure 1A, Chen et al. (2010) rapidly prepared large-area concave MLAs on quartz glass by this technique. Tightly stacked rectangular and hexagonal MLAs with diameters less than 100 μm were successfully fabricated in less than 3 h. Not only in planar transparent hard and brittle materials, femtosecond laser-assisted wet etching strategy also demonstrates flexibility in adjusting the shape and depth of structures. Du et al. (2012) prepared a honeycomb concave MLAs on a 3 mm diameter glass column. Hu et al. (2018) have successfully fabricated built-in microlenses in three-dimensional glass microfluidic channels. A wide range of continuous tuning by filling the channel with media with different refractive indices, which opens up new avenues for applications including biomedical imaging and sensing. Inspired by insect compound eyes, Wang et al. also successfully prepared glass infrared artificial compound eyes by this strategy. It has excellent infrared thermal imaging performance and a 60%–70% transmission rate (Wang et al., 2022b).

Wet etching of crystalline materials is usually based on crystal orientation etching, which leads to distortion and pattern distortion of the structure (Deng et al., 2016). Therefore, it is not suitable for the preparation of high-quality bending devices. Dry etching technique is using the gas plasma to physically bombard and chemically react simultaneously with the etched material under the action of electric field in a low vacuum environment, which can avoid this problem to a large extent.

Liu et al. (2017) first proposed a dry etching-assisted femtosecond laser modification technique for processing hard materials, as shown in Figure 1B. They successfully prepared uniform, square and hexagonal MLAs with high-quality focusing and imaging capabilities on fused silica, gallium arsenide, silicon carbide, and diamond. This technology significantly improves processing efficiency (Liu et al., 2019b). More importantly, the technology is compatible with integrated circuit manufacturing processes and has significant application potential in the field of device integration. In addition, the technique enables the preparation of compound eye structures on curved sapphire. Compared with direct laser ablation, the processing efficiency of this technique can be improved by more than two orders of magnitude. Due to its high hardness and thermal stability, the sapphire concave compound eyes can be used as high-temperature and hard-cast templates.

To further solve the problem of difficulty in preparing deep structures, Zheng et al. (2021) achieved grating structures with adjustable period, duty cycle, and height on the sapphire surface by successively combining wet etching and dry etching processes assisted by femtosecond laser processing. The roughness of the sapphire grating structure was reduced from 78 nm (after laser direct writing) to 7 nm (after dry etching). Liu et al. realized the preparation of sapphire infrared windows with double-sided subwavelength pyramidal structure arrays using femtosecond laser-assisted etching strategy (Liu X.-Q et al., 2022). Notably, by introducing a sacrificial layer protection strategy, the competing problems of surface damage and internal damage during deep processing by inside-out femtosecond laser were solved. This provides a new idea for preparing transparent hard and brittle materials for micro/nanostructure preparation.

### Liquid-assisted femtosecond laser ablation strategy

Liquid-assisted femtosecond laser ablation is another operative method to achieve high-precision true 3D processing of transparent hard and brittle materials. The femtosecond laser pulse is tightly focused at the intersection of material and liquid. The extremely high peak power of the laser focus causes the ablation of materials and pierces the liquid to produce laser cavitation. The shock wave generated by the expanding plasma plume and bubble collapse carries the ablated debris away from the surface, enabling real-time cleanup of debris during the ablation process. High-precision 3D processing of transparent hard and brittle materials is achieved through continuous controlled layer-by-layer material removal.

By comparing femtosecond laser processing in air and liquid, researchers found that liquid-assisted femtosecond laser ablation has better structural surface quality and higher processing resolution, with 1/3 to 1/2 reduction in ablation features (Cao et al., 2018). Sun et al. (2019) reported that liquid-assisted femtosecond laser processing reduced the ablation threshold of fused silica from 2.22 to 1.02 J/cm^2^. Since the femtosecond laser induces bubbles in the liquid after forming the plasma, different bubbles cause different impact pressures. Therefore, the ablation threshold reduction is closely related to the liquid with different properties. In addition, Wang et al. also prepared high-quality silicon carbide through-hole arrays without cracks and heat-affected zones by water-assisted femtosecond laser ablation technique, which is essential for the high-quality processing of silicon carbide electronic devices (Wang et al., 2021).

Liquid-assisted laser ablation can also reduce the thermal effects generated during laser processing. However, suspended debris and liquid flow can reduce the transmission stability of the femtosecond laser beam, resulting in less efficient energy transfer. For this reason, researchers proposed to process the back surface of materials to solve the above problem, i.e., laser-induced backside wet etching (LIBWE). This is the primary method for processing microstructures of transparent hard and brittle materials. Actually, LIBWE is mainly carried out through nanosecond laser pulses. Femtosecond laser pulses can easily lead to a liquid breakdown. However, considering the low heat affected zone, high resolution, and high surface quality, some studies still use femtosecond laser pulses. In this way, microstructures can be prepared in transparent hard and brittle materials with higher precision. For example, Ehrhardt et al. discovered two different laser-induced periodic surface structures (LIPSS) on SiO_2_ surfaces for the first time by LIBWE. This also provides additional data to discuss the origin of high spatial frequency LIPSS formation (Ehrhardt et al., 2018). Tan et al. (2019) successfully prepared 3D microchannels on glass by LIBWE. They used simultaneous spatial-temporal focusing to avoid the nonlinear self-focusing in the conventional focusing process. Seo et al. (2020) prepared glass microchannels using LIBWE. Based on this, combined with the laser-induced chemical liquid phase deposition method for rapid deposition of copper.

In addition, Hua et al. (2022) prepared different types of sapphire microlenses by cavitation-assisted femtosecond laser ablation technique. As shown in Figure 1C, the external profile of the prepared spherical microlenses is consistent with the theoretical design. Moreover, the further prepared square and hexagonal sapphire convex MLAs that can reach a fill factor of 100%, which is difficult to achieve by other techniques. This technique has also achieved the high-precision preparation of sapphire micro-optical components such as holographic diffraction elements, vortex light generators, and 3D artificial compound eyes.

Liquid-assisted laser ablation technology is based on an ablation processing mode and is suitable for any materials. The flow of the liquid eliminates the effect of debris during processing and reduces the thermal effects generated during processing. At the same time, the auxiliary cavitation kinetic process makes the machining more flexible and stable. However, achieving control of the process is complex, and the bubbles’ persistence, damage resistance, and mobility can simultaneously determine the final structural properties.

### Femtosecond laser combined with annealing strategy

The annealing process is a traditional heat treatment technique. It can effectively eliminate the residual stress inside the material after laser processing and reduce the surface roughness of the material (Maia et al., 2021).

For glass materials, annealing of the laser-ablated structure allows the glass surface to start softening first. The softened surface produces a slight localized reflux under gravity and surface tension, resulting in a smoothness similar to a liquid surface. After cooling, the surface is again transformed into a glassy substance, while the surface quality is substantially improved. Seuthe et al. (2017) analyzed the structural relaxation of multi-component lithium silicate glass after femtosecond laser ablation combined with annealing using Raman spectroscopy. The results indicated that femtosecond laser-induced structural modifications were closely related to local changes in the refractive index of the materials. Subsequently, Sala et al. (2021) characterized the improvement in surface quality after thermal annealing, achieving a reduction in roughness from 49 nm to 19 nm. The reduction in roughness was demonstrated by the mirror imaging properties before and after thermal annealing. High-quality glass micro-optical elements were successfully prepared using the smoothing properties of this strategy (Lin et al., 2009; Wang et al., 2022). Compared with direct laser ablation, the annealing-assisted femtosecond laser processing technique can significantly improve processing efficiency and surface quality.

In addition, CO_2_ laser annealing also can be combined with femtosecond laser processing. The surface layer of the material is heated under the irradiation of a CO_2_ laser. Due to the surface tension of the material, the viscosity is reduced, thus improving the surface quality. Schwarz et al. (2018) prepared high-quality axicon for generating quasi-Bessel beams in fused silica by combining femtosecond laser ablation and CO_2_ laser polishing processes. This strategy provides the possibility for rapid prototyping of glass elements, even 3D optical elements with complex free-form surfaces. Microlens arrays with high profile accuracy and low roughness have also been successfully fabricated by this strategy (Figure 1D). It is also confirmed that femtosecond laser ablation combined with CO_2_ laser annealing is suitable for preparing complex optical geometry (Schwarz et al., 2020; Schwarz et al., 2021).

For crystal materials, laser ablation produces a rough amorphous layer. However, the main body of the structure remains in the single crystal state. By using a temperature higher than the softening point of the amorphous state and lower than the melting point of the crystal, it is possible to give the amorphous layer enough internal energy to soften and volatilize without changing the structure of the crystal body (He et al., 2013). Xu et al. (2013) prepared optical waveguides with low cladding and bilinear structure in LiTaO_3_ crystals using femtosecond laser ablation combined with annealing. After the thermal annealing process, the propagation loss of the cladding waveguide was reduced and its transmission loss was minimized to 0.38 dB/cm. He et al. (2016) observed grating regeneration during annealing and prepared a negative refractive index fiber Bragg grating with excellent performance. In addition, the stresses accumulated during laser processing can be eliminated by annealing treatment. Based on this, the crack-free 3D microstructure is also realized.

The thermal annealing process has become a common material treatment process. However, annealing temperature and time need to be strictly controlled for different materials. For example, too long an annealing time can lead to the deformation of glass body material. Structural deformation and surface quality need to be measured. For crystalline materials, the smoothing effect of annealing treatment on the material surface is relatively weak, requiring better surface preparation during laser processing.

## Conclusion and outlook

We analyze the problems of low processing accuracy and poor structural surface quality as well as the severe damage to the material surface caused by femtosecond laser ablation technology. The research progresses of femtosecond laser hybrid processing strategies of transparent hard and brittle materials in recent years are reviewed. Hybrid processing strategies has become a new direction for micro-nano processing. For instance, femtosecond laser-assisted etching strategy can improve processing quality. However, as a point-by-point direct writing technology, the processing efficiency of femtosecond laser ablation can not meet the high requirements, and new auxiliary strategies need to be explored to improve the processing efficiency, such as parallel laser micro-nano processing technology. In addition, the combination of multiple technologies in order to achieve the integration of micro/nanodevices needs to be further investigated. In conclusion, femtosecond laser hybrid processing strategies play an increasingly significant role in the preparation and application of micro/nano devices for transparent hard and brittle materials. With the further exploration of femtosecond laser micro-nano processing technology, the preparation of micro/nano devices with arbitrary shape, low roughness and high resolution can be realized, which promote the development and accelerate the industrialization process of femtosecond laser micro-nano processing in aerospace, biomedical, information technology, new energy, new materials and other industries.
